# The AMPK–mTOR Pathway Is Inhibited by Chaihu Shugan Powder, Which Relieves Nonalcoholic Steatohepatitis by Suppressing Autophagic Ferroptosis

**DOI:** 10.1155/2024/4777789

**Published:** 2024-10-28

**Authors:** Zheng Liang, Dajin Pi, Jianwei Zhen, Haizhen Yan, Chuiyang Zheng, July Liang Chen, Wen Fan, Qingliang Song, Jinyue Pan, Dongdong Liu, Maoxing Pan, Qinhe Yang, Yupei Zhang

**Affiliations:** Jinan University, Guang Zhou 510632, China

**Keywords:** autophagic ferroptosis, Chaihu Shugan powder, naringenin, nonalcoholic steatohepatitis, Saikosaponin A

## Abstract

Nonalcoholic steatohepatitis (NASH) is the advanced stage of nonalcoholic fatty liver disease (NAFLD), which is distinguished by the accumulation of fat in the liver, damage to liver cells, and inflammation. Chaihu Shugan powder (CSP), a renowned traditional Chinese medicine (TCM) blend extensively utilized in China to address liver disease, has demonstrated its efficacy in reducing lipid buildup and effectively combating inflammation. Hence, the primary objective of this research is to examine the impacts and possible mechanisms of CSP on NASH through assessments of liver histopathology, lipidomic analysis, and gene expression. To induce a mouse model of NASH, we employed a diet which deficient in methionine and choline, known as methionine–choline deficient (MCD) diet. Initially, we examined the impact of administering CSP to NASH mice by assessing the levels of serum and liver indicators. We found that CSP was able to reduce lipid buildup and inflammation in mice. In addition, a total of 1009 genes exhibited enrichment in both the autophagy and ferroptosis pathways. The liver protein levels of Adenosine monophosphate-activated protein kinase–mammalian target of rapamycin (AMPK–mTOR)-mediated autophagy and ferroptosis markers, such as p-AMPK*α*/AMPK*α*, p-mTOR/mTOR, Beclin-1, microtubule associated protein 1 light chain 3 gamma (LC3), p62 (sequestosome 1 [SQSTM1/p62]), Kelch-like ECH-associated protein 1 (KEAP1), nuclear factor erythroid 2-related factor 2 (Nrf-2), ferritin heavy chain 1 (FTH1), and glutathione peroxidase 4 (GPX4), were restored by CSP. Furthermore, our findings indicated that the suppression of autophagy had a repressive impact on the occurrence of ferroptosis in the mouse model, indicating that autophagy activation likely plays a role in mediating ferroptosis in NASH.

## 1. Introduction

Nonalcoholic steatohepatitis (NASH), an intense type of nonalcoholic fatty liver disease (NAFLD), is now recognized as a major worldwide health concern [1]. NASH, which is marked by liver steatosis, hepatocyte damage, and inflammation, is not only a liver disorder but also a manifestation of the current worldwide crisis in metabolic health. Epidemiologically, NAFLD affects approximately one-quarter of the global population, with a significant proportion progressing to NASH [2, 3]. Recent estimates suggested that NASH may affect up to 1.5%–6.45% of the global population, translating to approximately 115 million to 500 million individuals [4]. With the rise of obesity and type 2 diabetes, it is expected that there will be corresponding increases in the prevalence rates of these conditions [5].

The clinical implications of NASH are profound. Lacking of timely treatment can lead to cirrhosis, hepatocellular carcinoma, and end-stage liver disease, further emphasizing the need for effective therapeutic strategies [6]. However, despite the disease's growing footprint and potential for severe outcomes, therapeutic options remain limited.

Given the pressing requirement for innovative treatments, traditional Chinese medicine (TCM) presents possible substitutes. Chaihu Shugan powder (CSP), a classic TCM formulation, has been employed extensively in China to treat liver diseases. Initial research has suggested that CSP is effective in decreasing lipid buildup and possesses strong anti-inflammatory characteristics [7–9]. However, the precise pharmacological mechanisms, particularly concerning NASH, remain largely uncharacterized.

Our previous studies utilized rat models to demonstrate that CSP may treat NAFLD through the p38 mitogen-activated protein kinase (MAPK) and JUN *N*-terminal kinase (JNK) pathways [10–12]. Additionally, we utilized network pharmacology to examine the nuclear receptor target and the associated constituents of CSP. Besides, we performed validation experiments on the NAFLD rat model to further identify the compounds and genes involved [13].

Our current investigation builds upon our prior studies. After conducting thorough research and foundational studies, our research group identified two crucial metabolites, namely, Saikosaponin A (SSa) and naringin (NGN). As stipulated by the Chinese Pharmacopoeia, SSa stands out as a primary constituent of *Radix Bupleuri*, the dry root of *chinense DC*. The main medicinal drug of CSP is *Bupleurum scorzoneri-folium* Willd., belonging to the *Apiaceae* family. The relevance of SSa is further accentuated by the literature, which highlights its potential role in modulating glucose and lipid metabolism, making it an intriguing compound for therapeutic explorations [14, 15]. Furthermore, through meticulous network pharmacology-based screenings, our research group identified NGN as another pivotal monomer within CSP [13]. Numerous studies have provided evidences for the importance of NGN, demonstrating its ability to reduce inflammation and lower lipid levels [16–18]. Such findings bolster the rationale of NGN being a potential therapeutic agent, especially in the context of metabolic disorders such as NASH. It is worth mentioning that these two metabolites obtained from CSP constituents have been shown to hinder autophagy and ferroptosis in various illnesses, although there is limited evidence of their role in autophagy and ferroptosis in liver diseases [19–22].

As a result, our research shifted focus to examining the underlying mechanisms of CSP by integrating SSa and NGN. To gain a comprehensive understanding of the roles of CSP in different aspects of NASH, we extensively examined liver tissue pathology, inflammation, lipidomic profiles, gene expression, autophagy pathways, and markers related to ferroptosis.

## 2. Materials and Methods

### 2.1. Drugs and Reagents

The CSP was acquired from Jiangyin Tianjiang Pharmaceutical Co., Ltd. The proportions of *Radix Bupleuri* dosage (Granule batch No. 21102283), *Citrus Reticulata* (Granule batch No. 21100763), *Chuanxiong Rhizoma* (Granule batch No. 20110483), *Paeoniae Radix Alba* (Granule batch No. 21100083), *Aurantii Fructus* (Granule batch No. 21090493), *Cyperi Rhizoma* (Granule batch No. 21091193), and *Radix Glycyrrhizae* (Granule batch No. 21101393) were 4:4:3:3:3:1. SSa (CAS 20736-09-8) was obtained from Shanghai Yuanye Technology Co., Ltd. NGN (CAS 480-41-1) was sourced from Shanghai Aladdin Biochemical Technology Co., Ltd., and polyene phosphatidylcholine capsules (PPCs) were supplied by Sanofi (Beijing) Pharmaceutical Co., Ltd. Kits for the determination of total cholesterol (TC), triglycerides (TGs), nonesterified fatty acids (NEFAs), high-density lipoprotein (HDL), low-density lipoprotein (LDL), alanine aminotransferase (ALT), aspartate aminotransferase (AST), superoxide dismutase (SOD), malondialdehyde (MDA), and glutathione peroxidase (GSH-PX) were obtained from Nanjing Jiancheng Bioengineering Institute, China. Enzyme-linked immuno sorbent assay (ELISA) kits were procured from MultiSciences (Lianke) Biotechnology Corporation Limited, based in Hangzhou, China. Ferrous Iron (Fe^2+^) Colorimetric Assay Kit was acquired from Elabscience Biotechnology Co., Ltd., Wuhan, China. Additionally, hematoxylin–eosin (HE) and Oil Red O (ORO) staining kits were purchased from Wuhan Servicebio Technology Co., Ltd. The antibodies were provided by Cell Signaling Technology (Beverly, MA, United States).

The L-amino acid diet (60 kcal% fat, 0.1% methionine, without adding choline, No. TP 36225MCD) was supplied by Trophic Animal Feed High-tech Co., Ltd. The control diet (methionine–choline sufficient (MCS) diet) is designed to match the methionine–choline deficient (MCD) diet, which is consisted of L-amino acid diet containing 10 kcal% fat, methionine, and choline, provided by Trophic Animal Feed High-tech Co., Ltd. (No. TP 36225MCS).

### 2.2. High-Performance Liquid Chromatography (HPLC) of CSP

To establish the presence of SSa and NGN in CSP, particles from a uniform batch of CSP were carefully measured following the proportions specified in the sections concerning animals and treatments. Approximately 0.5 g of the CSP was placed into a sealed conical flask, to which 10 mL of 70% methanol solution was added. Afterward, the combination was subjected to ultrasonic extraction for a duration of 30 min, followed by filtration using a membrane with a pore size of 0.45 μm, after which it was ready for analysis. Then, the CSP solution was diluted in a gradient ranging from 0.1 to 1000 μg/mL. The concentration of SSa, a conventional medication, was diluted in a range of 1–100 parts per million (ppm). We utilized a HPLC setup (1260, Agilent) equipped with an Agilent C18 chromatographic column measuring 4.6 × 250 mm and containing particles 5 μm in size. The mobile phase consisted of acetonitrile (solvent A) and purified water (solvent B), flowing at a rate of 1.0 mL/min. Gradient elution was performed as follows: 0–50 min, linear gradient from 25% to 90% solvent A; 50 to 55 min, 90% solvent A; and 55 to 57 min, linear gradient from 90% to 25% solvent A. The parameters were established with a column temperature of 25°C, a flow rate of 1 mL/min, and a sample injection volume of 10 μL. We detected the reference compounds at a monitoring wavelength of 210 nm. NGN was diluted in a gradient from 1 to 100 ppm. An Agilent HPLC system (Model 1260) was employed, featuring a chromatographic column (Agilent C18) with dimensions of 4.6 × 150 mm and a particle size of 5 μm. The mobile phase consisted of a water–methanol (1 : 1) solution containing 1% formic acid. The column temperature was set at 25°C, with a flow rate of 1 mL/min and a sample volume of 10 μL for introduction. Compounds that were genuine (reference) were identified at a wavelength of 320 nm.

### 2.3. NASH Model Establishment and Treatment

Five-week-old male C57BL/6J mice were obtained from Beijing HFK Biochemical Technology Co., Ltd. This study was conducted at the Institute of Laboratory Animal Science, Jinan University (Guangzhou, China), accordance with approved experimental protocols (Animal Ethics No. IACUC-20220114-06). The mice were randomly divided into groups and housed in a controlled environment with regulated temperature and humidity, following a 12-h light/dark cycle. During the research, all mice had free access to food and water.

In the first experiment, mice were divided into six groups based on their weight: (I) the Control group, which received an MCS diet for 3 weeks; (II) the NASH group, which received an MCD diet for 3 weeks; (III) the NASH + CSP group, which received CSP (containing 9.6 g/kg crude herbs) in addition to an MCD diet for 3 weeks; (IV) the NASH + SSa group, which received SSa (10 mg/kg/day) in addition to an MCD diet for 3 weeks; (V) the NASH + NGN group, which received NGN (100 mg/kg/day) in addition to an MCD diet for 3 weeks; and (VI) the NASH + PPC group, which received PPC (120 mg/kg/day) in addition to an MCD diet for 3 weeks.

In the second experiment, the mice were divided into three groups based on their weight: (I) the Control group, which received a diet containing enough methionine–choline for 3 weeks; (II) the NASH group, which received a diet deficient in methionine–choline for 3 weeks; (III) the NASH + 3-MA group, which received intraperitoneal injections of 3-MA (15 mg/kg/day, dissolved in DMSO; MedChemExpress) in addition to an MCD diet for 3 weeks.

### 2.4. Biochemical Analysis

After 12-h of fasting period, the mice were anesthetized by intraperitoneal injection of pentobarbital (50 mg/kg). Blood samples were reserved and centrifuged at 4°C and 3600 rpm for 15 min to extract serum. Serum levels of ALT, AST, HDL, and LDL were measured by using assay kits according to the manufacturers' instructions. Besides, mice livers from each group were weighed, homogenized, and then centrifuged to extract the supernatants. The concentrations of TC, TG, and NEFA of the liver homogenates were tested with corresponding detection kits.

### 2.5. Histopathological Examination

Liver tissues for histopathological examination were reserved after euthanasia and fixed with 4% paraformaldehyde. Afterward, tissues were encased in paraffin for long-term preservation. To estimate the overall histopathological discrepancy, the paraffin samples were sliced into sections of 5 µm thickness and HE staining was performed. To further observe the lipid accumulating situation, ORO was applied to dye on the samples' neutral lipids for assessing. In addition, immunofluorescence (IF) staining was performed with F4/80, CD68 (differentiation cluster 68), and CD206 (differentiation cluster 206) antibodies aimed to assessing macrophage infiltration. Besides, dihydroethidium (DHE) staining was performed according to the manufacturer's instructions to detect reactive oxygen species (ROS) in liver tissue. The ultrastructure of liver tissue was observed by transmission electron microscopy (TEM). We primarily focused on observing the changes of cellular structures of liver cell, distribution of lipid droplets, generation of autophagosomes, and construction of organelles, such as mitochondria and endoplasmic reticulum. Different magnifications of section images were captured at both overview and detailed views.

### 2.6. Cytokine Analysis Using ELISAs

ELISA kit was used to detect the levels of interleukin (IL)-1*β*, IL-6, and tumor necrosis factor (TNF)-*α* in liver tissues. All the experiments were repeated for six times, and pg/mL was used to express cytokine levels.

### 2.7. Detection of Oxidative Stress Indexes

Liver tissues were homogenized and the supernatants were extracted. Liver SOD, MDA, GSH, and Fe^2+^ levels were determined according to kit instructions. The experiments were repeated six times.

### 2.8. Liver Lipidomic Analysis

Lipid composition was analyzed by ultra-high-performance liquid chromatography (UHPLC) system and high-resolution mass spectrometry. Lipid metabolites are considered different-altereds (DALs) meeting the following criteria: projected variable importance (VIP) greater than 1, *p*-value less than 0.05, fold change (FC) equal to or greater than 2 or equal to or less than 0.5.

### 2.9. Liver Transcriptomic Analysis

After assessing the quality and quantity of extracted ribonucleic acid (RNA) using the NA Nano 6000 Assay Kit of the Bioanalyzer 2100 system (Agilent Technologies, CA, United States), only samples with an RNA integrity value (RIN) greater than 7.0 were used for subsequent analysis.

### 2.10. Western Blot Analysis

Protein bands were detected by enhanced chemiluminescence after electrophoresis, membrane transfer, and reaction with primary and secondary antibodies, and band intensity was measured by image analysis software.

### 2.11. Statistical Analysis

GraphPad Prism 9.0 software was utilized for all statistical computations. The data are displayed as the average plus the standard deviation. *⁣*^*∗*^*p* < 0.05, *⁣*^*∗∗*^*p* < 0.01, *⁣*^*∗∗∗*^*p* < 0.001, and *⁣*^*∗∗∗∗*^*p* < 0.0001.

## 3. Results

### 3.1. CSP, SSa, and NGN Reduced Hepatic Steatosis and Hepatocyte Injury in Mice

**Figure 1:**
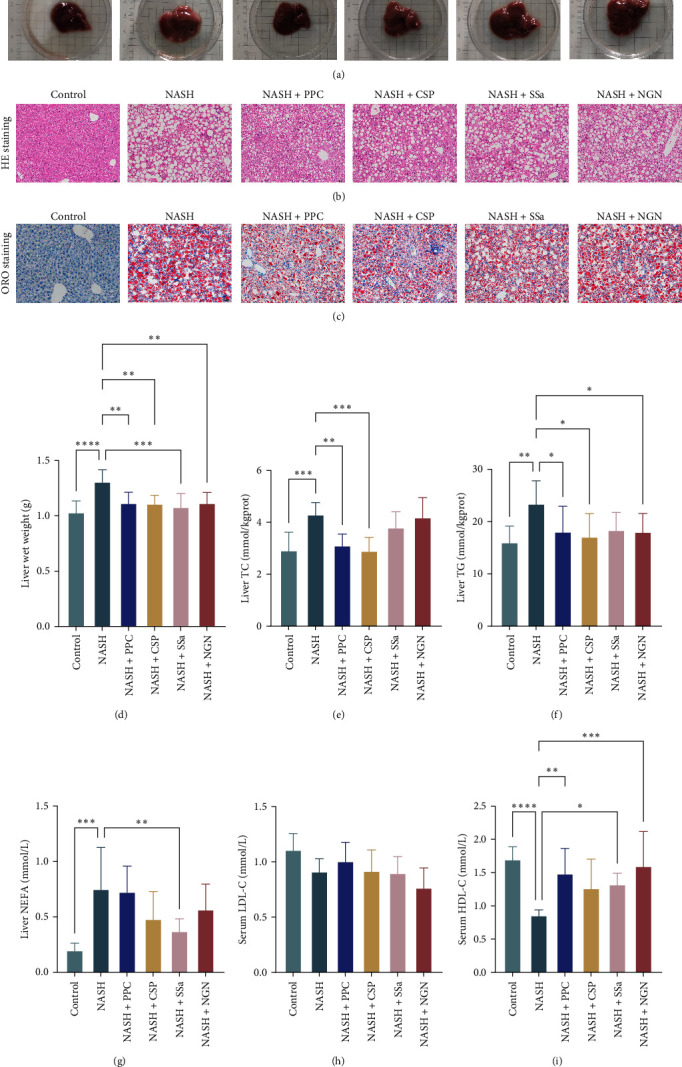
CSP, SSa, and NGN demonstrated therapeutic effects in the NASH model (*n* = 6). (a–c) Representative liver and histological images of liver tissue sections stained with HE and Oil Red O (scale bar: 50 µm; original magnification, ×200). (d) Hepatic weight across various mouse groups. (e–g) Measurements of TC, TG, and NEFA concentrations in the liver specimens from each group. (h and i) Serum concentrations of HDL-C and LDL-C. CSP, Chaihu Shugan powder; HDL, high-density lipoprotein; HE, hematoxylin–eosin; LDL, low-density lipoprotein; NASH, nonalcoholic steatohepatitis; NEFA, nonesterified fatty acid; NGN, naringin; PPC, polyene phosphatidylcholine capsule; SSa, Saikosaponin A; TC, total cholesterol; TG, triglycerides. *⁣*^*∗*^*p* < 0.05, *⁣*^*∗∗*^*p* < 0.01, *⁣*^*∗∗∗*^*p* < 0.001, and *⁣*^*∗∗∗∗*^*p* < 0.0001.

Research indicates that PPC is a liver-protective medication that has been extensively utilized in clinical settings and is capable of improving liver function and blood lipid levels in mice with NASH. To validate the inhibition of NASH advancement due to the actions of CSP, SSa, and NGN (as shown in Supporting Information 2, SSa and NGN have been proven to be active components of CSP), we employed PPC as a reference for comparison. The liver tissues stained with HE were examined histologically and revealed significant steatosis, inflammation, and fibrosis in the NASH mice. However, the mice treated with CSP, SSa, and NGN exhibited notable reductions in these conditions (Figure 1a,b). The NASH mice exhibited a significant increase in lipid droplets, as revealed by ORO staining, suggesting the presence of lipid accumulation. In contrast, the use of CSP, SSa, and NGN significantly reduced the proportion that tested positive by ORO staining, suggesting a decrease in the accumulation of lipids in the liver (Figure 1c).

After the development of NASH, mice showed a notable increase in liver weight, suggesting the presence of hepatic steatosis. However, the administration of CSP, SSa, and NGN resulted in a notable decrease in liver weight, indicating a defensive impact against hepatic steatosis induced by NASH (Figure 1d). Dysregulated lipid metabolism in NASH mice was indicated by significantly elevated levels of TC, TG, and NEFA in the liver, while the administration of CSP, SSa, and NGN resulted in notable decreases in hepatic TC, TG, and NEFA concentrations, indicating enhanced lipid metabolism (Figure 1e–g). Crucially, the serum levels of HDL-C and LDL-C showed no notable disparity among the groups, affirming the effective establishment of the MCD-induced NASH model (Figure 1h,i).

### 3.2. CSP, SSa, and NGN Extensively Modulated the Hepatic Lipid Profile

**Figure 2:**
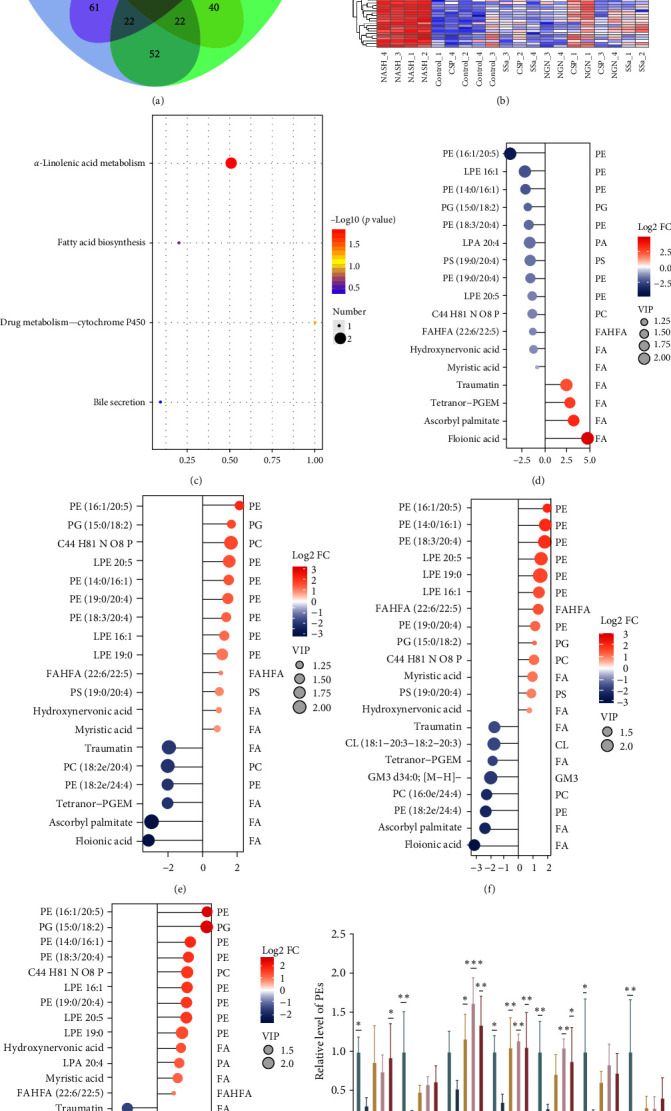
CSP, SSa, and NGN exert widespread effects on hepatic lipid metabolism. We performed a lipidomics analysis of murine hepatic tissue samples (*n* = 4). (a and b) A Venn diagram of the DALs was constructed, and the results were clustered to create a heatmap. (c) KEGG enrichment was performed on the DALs, and a bubble chart was created. (d) Matchstick plot of DALs for NASH in comparison to the Control group. (e) Matchstick plot of DALs for CSP in comparison to the NASH group. (f) Matchstick plot of DALs for SSa in comparison to the NASH group. (g) Matchstick plot of DALs for NGN in comparison to the NASH group. (h) Presentation of the results of PE reversal by CSP, SSa, and NGN. CSP, Chaihu Shugan powder; DALs, different-altered; FC, fold change; NASH, nonalcoholic steatohepatitis; NGN, nonesterified; PE, phosphatidylethanolamine; SSa, Saikosaponin A; VIP, variable importance. *⁣*^*∗*^*p* < 0.05, *⁣*^*∗∗*^*p* < 0.01, *⁣*^*∗∗∗*^*p* < 0.001, and *⁣*^*∗∗∗∗*^*p* < 0.0001.

To validate the impact of CSP, SSa, and NGN on the metabolism of liver lipid, we performed a lipidomic analysis to detect changes in the liver lipid profiles in the Control, NASH, CSP, SSa, and NGN groups. The lipidomic analysis identified the shared DALs (Figure 2a) among the control, experimental, and three treatment groups. Subsequently, we conducted cluster analysis (Figure 2b) and kyoto encyclopedia of genes and genomes (KEGG) (Figure 2c) enrichment analysis. As shown in Figure 2c, we observed that the differentially regulated lipids were mainly clustered in the pathways related to fat metabolism. Additionally, the drug metabolism–cytochrome P450 pathway was also implicated, which has been linked to ferroptosis in other reports.

Moreover, the quantities of phosphatidylethanolamines (PEs), such as PE (16:1/20:5), LPE 16:1, LPE 19:0, PE (19:0/20:4), PE (18:3/20:4), PE (14:0/16:1), and LPE 20:5, were notably reduced as a result of MCD consumption but were subsequently restored by the administration of CSP, SSa, and NGN (Figure 2d–g). Simultaneously, although CSP, SSa, and NGN were all able to reverse the downregulation of MCD-induced PEs, their specific effects of reversal varied (as shown in Figure 2h).

These results indicate that CSP, SSa, and NGN might contribute to the prevention of NASH by influencing the lipid composition in the liver. The comprehensive control of liver fats may play a role in the observed enhancements in liver structure and operation, further endorsing the potential healing advantages of CSP, SSa, and NGN in treating NASH.

### 3.3. CSP, SSa, and NGN Alleviated Hepatic Inflammation Linked to NASH

**Figure 3:**
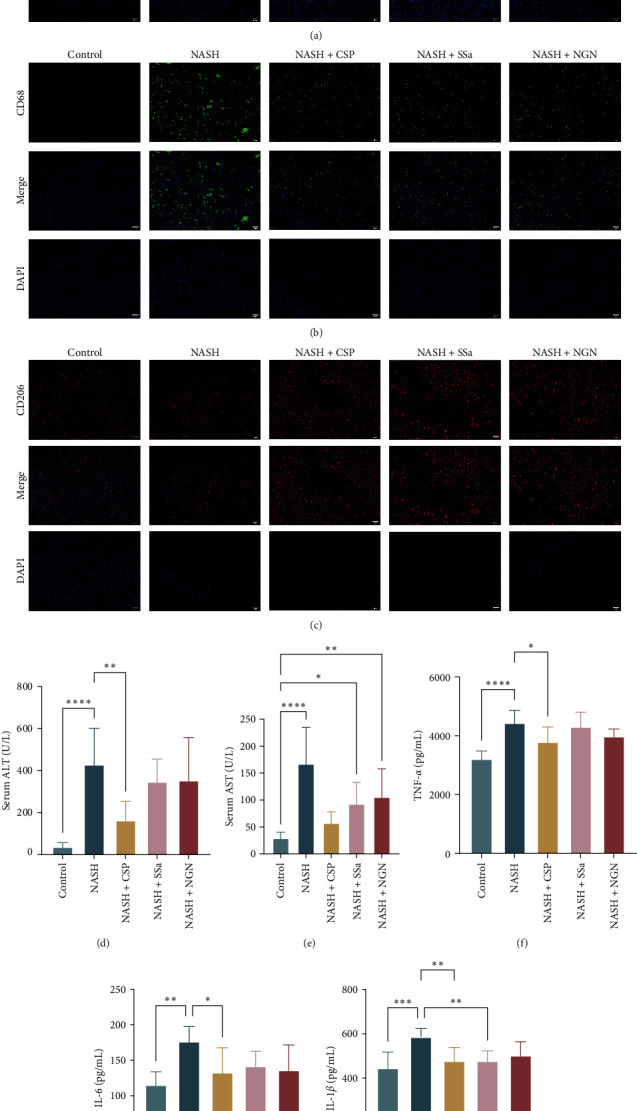
The development of steatohepatitis in mice fed an MCD diet can be reduced by treating them with CSP, SSa, and NGN. (a) Micrographs depicting F480 staining within hepatic sections, annotated with a scale bar at 50 µm and captured at the original 200-fold magnification (*n* = 3). (b and c) Micrographs depicting staining for CD68 and CD206 within hepatic sections, annotated with a scale bar at 50 µm, and captured at the original 200-fold magnification (*n* = 3). (d and e) Serum AST and ALT levels. The findings are displayed as the average ± standard deviation (*n* = 6). (f–h) Levels of TNF-*α*, IL-6, and IL-1*β* in the liver. The findings are displayed as the average ± standard deviation (*n* = 6). ALT, alanine aminotransferase; AST, aspartate aminotransferase; CSP, Chaihu Shugan powder; IL, interleukin; MCD, methionine–choline deficient; NGN, nonesterified; SSa, Saikosaponin A; TNF-*α*, tumor necrosis factor-*α*. *⁣*^*∗*^*p* < 0.05, *⁣*^*∗∗*^*p* < 0.01, *⁣*^*∗∗∗*^*p* < 0.001, and *⁣*^*∗∗∗∗*^*p* < 0.0001.

Administering CSP, SSa, and NGN can alleviate the development of steatohepatitis in MCD-fed mice. IF staining of F4/80 was performed to evaluate hepatic macrophages (Figure 3a).

Our results revealed that the livers of NASH mice fed a 3-week MCD diet displayed the highest fluorescence intensity for F4/80. However, treatment with CSP, SSa, and NGN significantly decreased the infiltration of hepatic macrophages in NASH mice. The fluorescence intensity of F4/80 staining confirmed these findings, indicating that the liver experienced a significant increase in macrophage recruitment after 3 weeks of administration of the MCD diet. However, the increase in macrophages was significantly lower in the drug-treated NASH group treated with CSP, SSa, and NGN than in NASH mice.

Based on the findings from other IF staining outcomes, CSP, SSa, and NGN administration resulted in elevated levels of CD206 (an M2 marker) compared to the NASH group; conversely, the expression of CD68 (an M1 marker) was lower (Figure 3b,c). We also observed increases in serum ALT and AST, along with hepatic levels of TNF-*α*, IL-6, and IL-1*β*, in mice that were fed an MCD diet. Significantly, the administration of CSP, SSa, and NGN effectively countered these modifications (Figure 3d–h).

### 3.4. The Expression Levels of Genes Involved in Adenosine Monophosphate-Activated Protein Kinase–Mammalian Target of Rapamycin (AMPK–mTOR)-Mediated Autophagy and Ferroptosis Promotion Were Regulated by CSP, SSa, and NGN Administration

**Figure 4:**
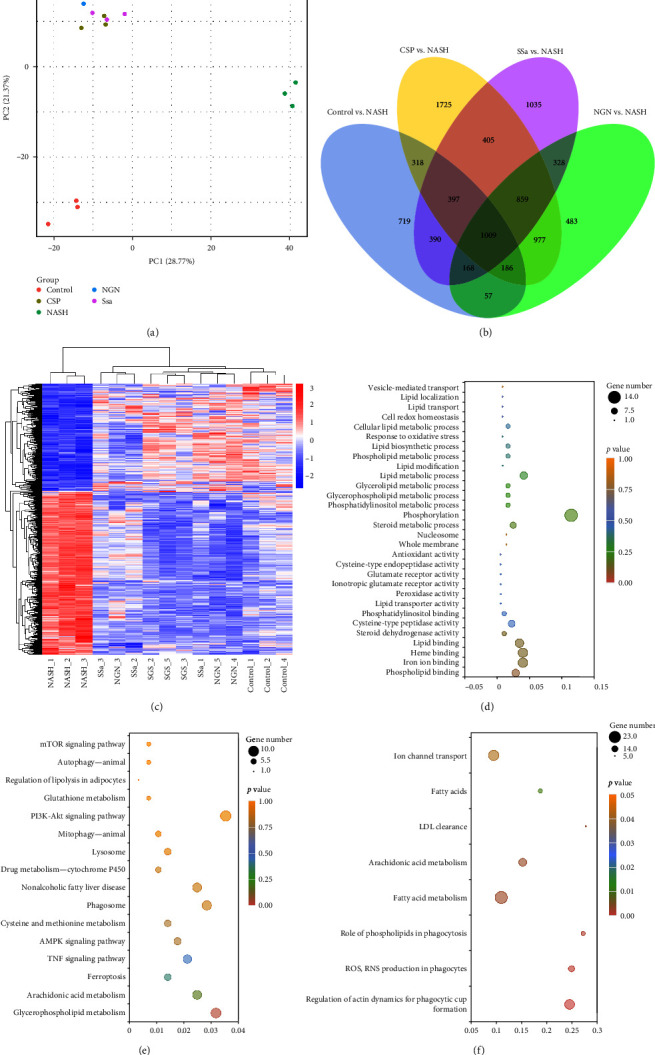
CSP, SSa, and NGN exert significant impacts on hepatic transcriptomic patterns. RNA-seq analysis was conducted on samples obtained from mouse liver tissue (*n* = 3). (a) A PCA model was constructed to define the gene phenotypes among all groups. (b and c) A heatmap was created by clustering the DEGs using a Venn diagram. (d–f) GO, KEGG, and Reactome enrichments were performed on the DEGs, and a bubble chart was created. CSP, Chaihu Shugan powder; DEGs, differentially expressed genes; NGN, naringin; PCA, principal component analysis; SSa, Saikosaponin A.

Transcriptomic analysis using RNA sequencing (RNA-seq) was employed to uncover the potential mechanisms through which CSP, SSa, and NGN influence lipid metabolism. As shown in Figure 4a–c, we discovered a common group of 1009 differentially expressed genes (DEGs) among the Control group, the NASH group, and the three treatment groups. These genes included Prkag2, Eef2k, Scd3, Fnip2, Sesn2, Cybb, Hmox1, Rab7b, Akt3, Atg13, Pik3cd, Ctsd, Lamp1, Ctsb, and Prkcd. Based on the results of the Gene Ontology (GO) analysis, the DEGs exhibited significant enrichment in lipid metabolism-related biological processes and iron ion binding in lipid metabolism, as shown in Figure 4d. Similarly, the analysis of KEGG pathways found numerous pathways that were significantly enriched, encompassing lipid metabolism, inflammation, oxidative stress, AMPK, mTOR, autophagy, and ferroptosis (Figure 4e). According to the reactome enrichment analysis, the DEGs exhibited notable concentrations in pathways related to phospholipid metabolism, autophagy, and ferroptosis (Figure 4f). The results suggest that CSP, SSa, and NGN may promote safeguarding effects against NASH by participating in the control of the expression of genes associated with crucial biological processes and pathways such as the AMPK–mTOR pathway, autophagy, and ferroptosis. We speculate that the expression levels of genes involved in AMPK–mTOR mediated autophagy and ferroptosis promotion are regulated by CSP, SSa, and NGN.

### 3.5. CSP, SSa, and NGN Impeded Liver AMPK/mTOR-Mediated Autophagy

**Figure 5:**
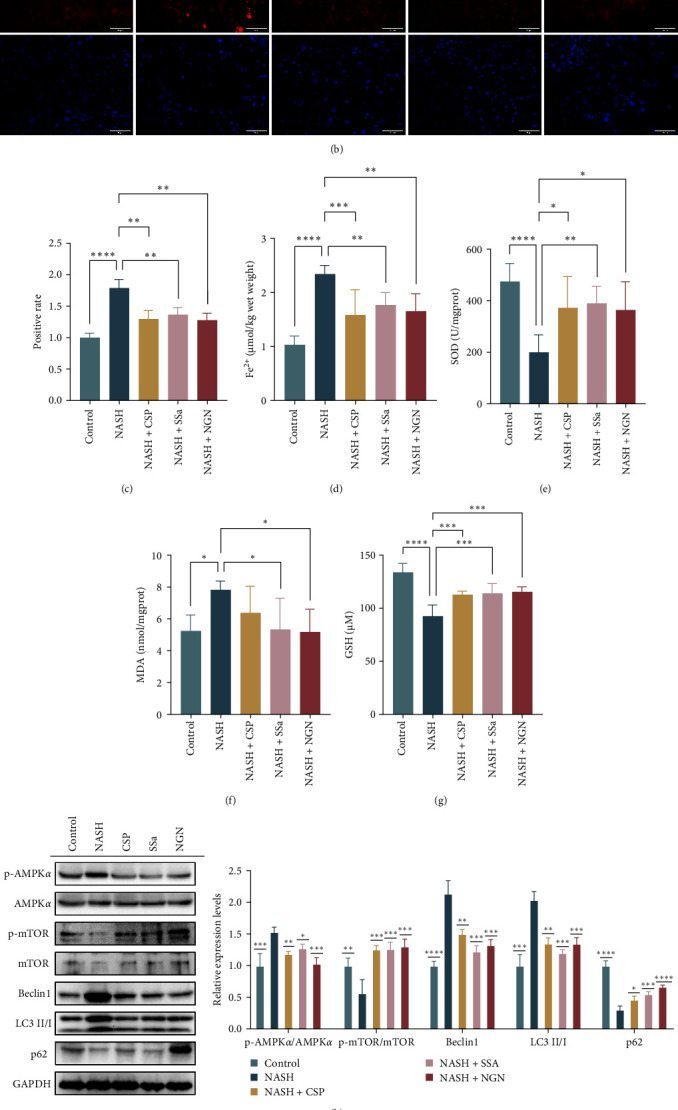
The progression of AMPK/mTOR-mediated autophagy and ferroptosis in MCD-fed mice can be mitigated by treatment with CSP, SSa, and NGN. (a) Observations of ultrathin liver sections were conducted using TEM (magnifications of 9700X and 23,000X). (b and c) ROS staining and relative expression levels (with a scale bar of 50 µm). (d–g) Liver Fe^2+^, SOD, MDA, and GSH levels. (h and i) Western blot analysis of the protein expression levels of p-AMPK*α*/AMPK*α*, p-mTOR/mTOR, Beclin1, LC3, p62, KEAP1, Nrf-2, FTH1, and GPX4, with a sample size of 3. The data are displayed as the average ± standard deviation. AMPK, Adenosine monophosphate-activated protein kinase; CSP, Chaihu Shugan powder; FTH1, ferritin heavy chain 1; GPX4, glutathione peroxidase 4; GSH, glutathione; KEAP1, Kelch-like ECH-associated protein 1; LC3, microtubule associated protein 1 light chain 3 gamma; MCD, methionine–choline deficient; MDA, malondialdehyde; mTOR, mammalian target of rapamycin; NGN, naringin; Nrf-2, nuclear factor erythroid 2-related factor 2; p62, sequestosome 1 (SQSTM1/p62); ROS, reactive oxygen species; SOD, superoxide dismutase; SSa, Saikosaponin A; TEM, transmission electron microscopy.

To clarify the impact of CSP, SSa, and NGN on hepatic autophagy in the context of NASH, we investigated the liver cell ultrastructure through TEM and analyzed the protein expression profiles of crucial autophagy-related components. In the liver cells of NASH mice, TEM revealed a notable buildup of autophagosomes, suggesting a disruption in the flow of autophagy. Nonetheless, CSP, SSa, and NGN administrations resulted in notable decreases in the quantity of autophagosomes, indicating that autophagic function was inhibited (Figure 5a).

Our findings were reiterated by assessing the expression levels of crucial autophagy-associated proteins (such asp-AMPK*α*/AMPK*α*, p-mTOR/mTOR, Beclin-1, microtubule associated protein 1 light chain 3 gamma (LC3), and p62 sequestosome 1 [SQSTM1/p62]), as shown in Figure 5h. In NASH mice, autophagy was upregulated, as evidenced by elevated levels of p-AMPK*α*/AMPK*α* and Beclin-1 and the reduced expression levels of p-mTOR/mTOR and p62. However, following treatment with CSP, SSa, and NGN, the expression levels of p-AMPK*α*/AMPK*α* and Beclin-1 decreased, while the expression levels of p-mTOR/mTOR and p62 increased, suggesting a decrease in autophagic activity. Additionally, a reduced conversion from LC3-I to LC3-II was observed, further corroborating the suppressive impact of these interventions on autophagy.

The results indicate that CSP, SSa, and NGN might contribute to the prevention of NASH by increasing the inhibition of hepatic autophagy to some extent.

### 3.6. CSP, SSa, and NGN Administration Hindered Ferroptosis to Decrease NASH

TEM analysis revealed that the MCD diet led to greater mitochondrial swelling and disappearance in NASH livers. Nonetheless, administration of CSP, SSa, and NGN protected the integrity of the mitochondrial structure.

Expanding on our existing approaches and to investigate the possible involvement of ferroptosis in the protective effects of CSP, SSA, and NGN against NASH, we measured the levels of ROS and Fe^2+^ related to ferroptosis in liver samples (Figure 5b–d). The liver cells of NASH mice exhibited notably increased ROS levels, as detected by ROS staining, suggesting the presence of oxidative stress and the possibility of ferroptosis. Nevertheless, the administration of CSP, SSa, and NGN resulted in notable decreases in ROS levels, indicating the suppression of ferroptosis.

To validate these findings, we conducted an analysis of the MDA levels (Figure 5f), which serve as an indicator of lipid peroxidation, as well as the activities of SOD and GSH-PX, two crucial enzymes involved in antioxidant defense (Figure 5e,g). Oxidative stress and potential ferroptosis were indicated by the observed increase in MDA level and the decreases SOD and GSH-PX activities in NASH mice. Nevertheless, the administration of CSP, SSa, and NGN significantly reduced MDA level while simultaneously boosting the functions of SOD and GSH-PX. These findings indicate an improvement in antioxidant protection and the suppression of ferroptosis.

Western blotting was also performed to examine the levels of Kelch-like ECH-associated protein 1 (KEAP1), nuclear factor erythroid 2-related factor 2 (Nrf-2), ferritin heavy chain 1 (FTH1), and glutathione peroxidase 4 (GPX4), which are crucial regulators of ferroptosis (Figure 5i). NASH mice showed an elevation in KEAP1 levels and decreased expression levels of Nrf-2, FTH1, and GPX4, suggesting the occurrence of ferroptosis. Nevertheless, the administration of CSP, SSa, and NGN notably inhibited KEAP1 expression. The inhibition of ferroptosis was further confirmed based on the expression of Nrf-2, FTH1, and GPX4.

These results indicate that CSP, SSa, and NGN might prevent NASH by partially inhibiting ferroptosis.

### 3.7. Intervention With 3-MA Validated That Autophagy Precedes Ferroptosis

**Figure 6:**
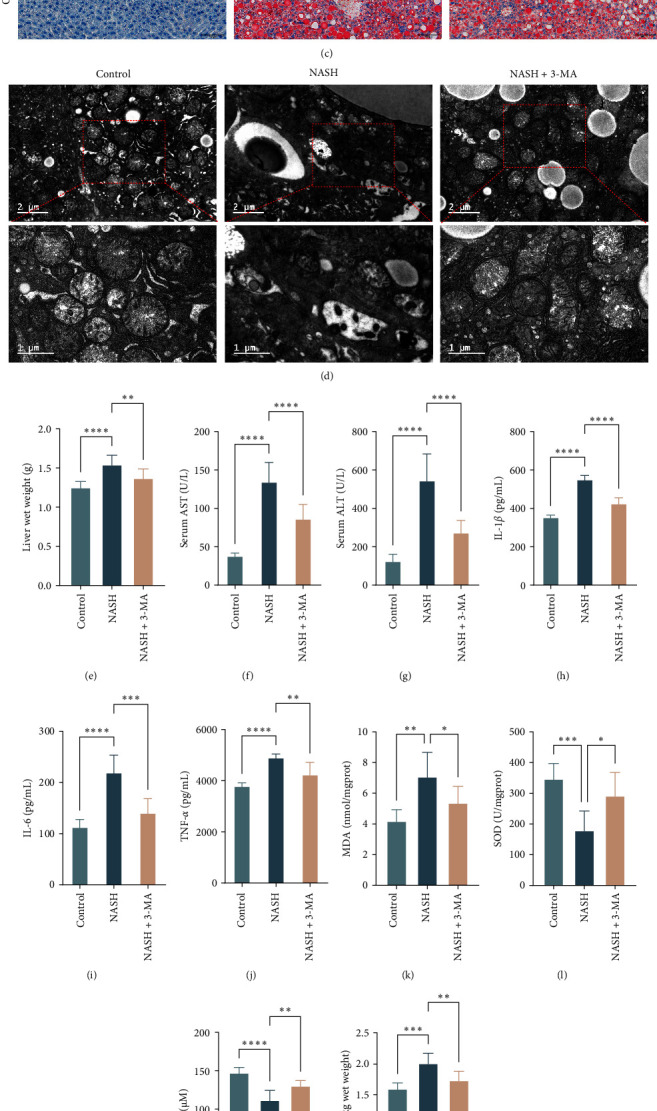
In the NASH model, 3-MA administration had beneficial effects. (a–c) Liver and histological images of liver tissue sections stained with HE and Oil Red O, respectively. The scale bar measures 100 μm; the original magnification was 200 times. (d) Examination of ultrathin hepatic sections via transmission electron microscopy at magnifications of 9700X and 23,000X. (e) Liver weights across the various mouse cohorts. (f and g) Serum AST and ALT levels. (h–j) Levels of IL-1*β*, IL-6, and TNF-*α* in the liver. (k–n) Liver Fe^2+^, SOD, MDA, and GSH levels. (o) Western blot analysis confirmed the protein expression levels of KEAP1, Nrf-2, FTH1, and GPX4 in the three sample groups. ALT, alanine aminotransferase; AST, aspartate aminotransferase; GPX4, glutathione peroxidase 4; GSH, glutathione; HE, hematoxylin–eosin; IL, interleukin; KEAP1, Kelch-like ECH-associated protein 1; MDA, malondialdehyde; NASH, nonalcoholic steatohepatitis; Nrf-2, nuclear factor erythroid 2-related factor 2; SOD, superoxide dismutase; TNF-*α*, tumor necrosis factor-*α*. *⁣*^*∗*^*p* < 0.05, *⁣*^*∗∗*^*p* < 0.01, *⁣*^*∗∗∗*^*p* < 0.001, and *⁣*^*∗∗∗∗*^*p* < 0.0001.

During our initial investigation, we discovered that autophagy had the ability to trigger ferroptosis, leading us to speculate that NASH may be a result of ferroptosis induced by autophagy. Therefore, we employed the autophagy inhibitor 3-MA in our intervention. Following the administration of the autophagy inhibitor, we observed notable reductions in both fatty degeneration and inflammation in the livers of MCD diet-fed mice (Figure 6a–c, e–j). Significantly, the increases in hepatic tissue Fe^2+^, SOD, MDA, and GSH levels caused by the MCD diet were counteracted by 3-MA administration (Figure 6k–n). Moreover, TEM revealed a notable enhancement in the cellular mitochondrial organization, providing compelling proof that the inhibition of autophagy effectively prevented ferroptosis (Figure 6d).

Additional confirmation was obtained through Western blot analysis (Figure 6o). When autophagy was inhibited, there were notable changes in the proteins linked to the ferroptosis pathway. In summary, in this NASH model, autophagy is the precursor of ferroptosis, and ferroptosis is the result of autophagy. The results indicate that autophagy causes NASH by inducing ferroptosis.

## 4. Discussion

This investigation represents an inaugural attempt to integrate both lipidomic sequencing and transcriptomics sequencing, offering a holistic, multidimensional perspective on CSP therapeutic modalities for NASH in mice.

The MCD diet-induced model for NASH is widely recognized for its capability to replicate the severe pathology of NASH [23]. Using this model, marked hepatocyte ballooning and lobular inflammation were observed through HE staining. Hepatocytes exhibited notable lipid accumulation, which was further emphasized by ORO staining. Treated mice displayed reduced liver weight, indicating a defensive impact against hepatic steatosis. The therapeutic potential of the compounds was highlighted by the reductions in TC, TG, and NEFA levels, as well as the increase in HDL, considering the protective function of HDL against cardiovascular diseases [24–26].

Utilizing lipidomic sequencing, we discovered that PE (16:1/20:5), lysophosphatidylethanolamine (LPE) 16:1, LPE 19:0, PE (19:0/20:4), PE (18:3/20:4), PE (14:0/16:1), and LPE 20:5 were significantly downregulated due to the MCD diet. However, these effects were subsequently reversed by the administration of CSP, SSa, and NGN. The liver experiences injury and mitochondrial dysfunction due to an occurrence known as “liptoxicity” which is caused by excessive lipid buildup [27, 28]. Very low-density lipoprotein (VLDL) is a key method by which the liver releases TGs into the bloodstream. The proper release of VLDL by hepatic cells is highly reliant on the production of phosphatidylcholine (PC). The conversion of PE into PC serves as an important source in this process [29, 30]. We speculate that these three drugs might have promoted an increase in PE content within the mice in some manner, thereby accelerating the process of VLDL entering the bloodstream from the liver, alleviating NASH. Throughout this procedure, we observed that CSP, SSa, and NGN administrations enhanced the levels of PE to different degrees, necessitating future investigation into the underlying mechanisms.

The progression of NASH largely depends on the inflammatory cascade. Histological assessment after CSP, SSa, and NGN interventions provided compelling evidence of their ability to mitigate inflammation. After intervention with CSP, SSa, and NGN, significant decreases in the levels of F4/80 and CD68 expression were noted, highlighting the diminished recruitment and activation of macrophages, which are key indicators of the regression of inflammation [31]. Additionally, the enhanced manifestation of CD206, which represents the regenerative M2 macrophage characteristic, also suggested a transition from primarily inflammatory to a healing liver setting [32]. On the serological front, the efficacies of CSP and its compounds were further evident. Posttreatment, there were significant reductions in the levels of ALT and AST, which are essential indicators of liver cell damage and inflammation. The decrease in transaminase levels provided evidence of decreased damage to liver cells and the potential of the compounds to protect the liver. Moreover, the simultaneous decreases in the expression levels of proinflammatory cytokines, such as IL-6, IL-1*β*, and TNF-*α*, highlighted the overall anti-inflammatory impact of CSP, SSa, and NGN.

Utilizing transcriptomic sequencing, we identified shared differentially expressed locis (DELs) among the groups, including the NASH group, resulting in effective clustering. Next, we attempted to identify potential biological processes and pathways through screening. Enrichment results suggested that the DEGs among the groups were enriched in autophagy and ferroptosis pathways. The TEM findings also indicated that CSP, SSa, and NGN decreased the quantities of autophagosomes and lysosomes while also alleviating mitochondrial damage. Our results suggest that CSP, SSa, and NGN alleviate NASH symptoms by inhibiting autophagy and ferroptosis.

The core of NASH involves the complex interactions between lipid metabolic dysregulation, inflammation, and various cell death pathways [33]. Ferroptosis is distinct from other types of cell death as it depends on lipid peroxidation caused by disruptions in iron and lipid metabolism [34, 35]. During the progression of NASH, excessive accumulation of lipids in hepatocytes can increase susceptibility to lipid peroxidation [36]. When combined with iron overload, this promotes the occurrence of ferroptosis. Subsequent cell death further exacerbates liver inflammation and damage, creating a vicious cycle that worsens NASH progression [37].

Previous studies have shown that autophagic flux in NASH is often greater than in NAFLD, thus excessive autophagy always accompanies the progression of NASH. On the other hand, excessive autophagy can increase hepatocyte inflammation and oxidative stress, exacerbating NASH. Therefore, they are causally related, leading to further damage to hepatocytes [38]. This observation is consistent with our experimental results. The connection between ferroptosis and autophagy is complex. Autophagy can control the balance of iron in the body by degrading cellular structures, thereby influencing the process of ferroptosis [39].

On the other hand, autophagy is regulated by numerous upstream signaling pathways. Enrichment results suggest that the AMPK-mTOR axis may play a key role in the treatment process of CSP, SSa, and NGN. This study reveals their complex roles in the regulation of autophagy. The AMPK pathway is a key regulator of cellular energy balance, while the mTOR pathway is crucial for cell growth and anabolic metabolism. These two pathways often act in an interactive manner, where AMPK activation inhibits mTOR signaling to promote catabolic processes that generate ATP [40]. In the specific context of NASH, the AMPK-mTOR pathway plays a role in various pathological processes such as lipid accumulation, inflammation, oxidative stress, and cell death. This process may involve the upregulation of autophagy in critical cells, which, if not regulated, could lead to autophagy-dependent ferroptosis. The alleviating effects of CSP, SSa, and NGN on NASH symptoms are primarily attributed to their influence on this particular pathway [41, 42].

In our study, CSP, SSa, and NGN inhibit autophagy by activating mTOR phosphorylation, ultimately preventing ferroptosis [43]. Ferroptosis is associated with exacerbated lipid peroxidation in NASH and is the result of the accumulation of lethal lipid ROS [39, 44]. Therefore, activating mTOR phosphorylation and simultaneously inhibiting AMPK phosphorylation through CSP helps to mitigate oxidative death caused by iron overload. Additionally, the inhibition of ferroptosis pathways through mTOR phosphorylation may not only stem from autophagy suppression but also from direct effects on ferroptosis regulators. Proteins that counter lipid peroxidation, such as GPX4, can function in an environment where mTOR phosphorylation is activated, thereby preventing ferroptosis [45].

Beclin1, as a fundamental component of autophagosome formation, plays a crucial role in autophagy. It accomplishes this process by binding with different proteins to generate autophagic precursors [46]. Our study results indicate that CSP, SSa, and NGN regulate autophagy by inhibiting AMPK phosphorylation and increasing mTOR phosphorylation expression, leading to reduced Beclin1 expression. The conversion of LC3 I to its PE-conjugated form LC3 II can serve as an indicator of autophagosome formation, indicating the progression of autophagy [47, 48]. p62, as a link between LC3 and ubiquitinated substances, regulates the degradation of ubiquitinated substances within autophagosomes [49–51]. In our study, we observed increased expression of Beclin1, decreased levels of p62, and increased conversion of LC3 I to LC3 II in the livers of NASH mice. After treatment with CSP, SSa, and NGN, the expression of Beclin-1 was significantly decreased and the expression of p62 was significantly increased, indicating a decrease in autophagy activity. Additionally, we observed a reduction in the conversion of LC3-I to LC3-II, further indicating that these interventions hindered the autophagic process.

Ferroptosis involves abnormal accumulation of iron ions and is a novel mode of programed cell death characterized mainly by the accumulation of iron-dependent toxic lipid peroxides [52]. The morphological characteristics of ferroptosis are significantly different from apoptosis, necrosis, and autophagy, primarily manifesting as reduced mitochondrial volume, increased membrane density, and reduced or absent mitochondrial cristae. The destruction of mitochondrial structure is a prerequisite for the ferroptosis cascade [35, 53]. Through TEM, we found characteristic mitochondrial structural changes in the livers of NASH mouse models, manifesting as the loss or significant reduction of mitochondrial cristae. With the intervention of CSP, SSa, and NGN, mitochondrial function was restored.

The core mechanism of ferroptosis lies in the harmful interaction between iron metabolism disorders and lipid peroxidation [54, 55]. Accumulation of ROS and Fe^2+^ can catalyze lipid peroxidation, leading to the accumulation of MDA, a precursor of cellular ferroptosis [56]. As a major organ for iron storage and lipid metabolism, abnormal iron metabolism and excessive accumulation of lipid peroxides in hepatocytes can lead to ferroptosis. Studies have shown that ferroptosis and lipid metabolism disorders play a critical role in the progression of NASH, and conversely, inhibiting ferroptosis can significantly reduce the severity of NASH [57]. Our study results indicate that administration of CSP, SSa, and NGN significantly reduced the levels of ROS, Fe^2+^, and MDA, indicating alleviation of ferroptosis and lipid peroxidation.

SOD and GSH-PX, as antioxidant enzymes, play important roles in combating oxidative stress and preventing lipid peroxidation. After treatment with CSP, SSa, and NGN, the levels of SOD and GSH-PX in the liver of mice increased, highlighting the antioxidant capabilities of CSP, SSa, and NGN, and further proving their inhibitory effects on ferroptosis. The basis of ferroptosis is lipid peroxidation, a process influenced by the cellular oxidative environment. The KEAP1-Nrf-2 signaling pathway plays a crucial role in maintaining cellular redox homeostasis. Under normal physiological conditions, KEAP1 guides the ubiquitination of Nrf-2, which is subsequently degraded by proteasomes. However, when exposed to an oxidative environment, the structure of KEAP1 changes, causing Nrf-2 to move into the nucleus. Once in the nucleus, Nrf-2 coordinates the transcription of many antioxidant genes, thereby enhancing the cell's ability to resist oxidative stress. The KEAP1-Nrf-2 axis indirectly mitigates ferroptosis through its antioxidant stress response. Additionally, the activation of Nrf-2 increases the expression of ferritin, which binds with free iron, thus affecting the regulation of ferritin on the Fenton reaction. The hydroxyl radicals produced by the Fenton reaction can damage lipids. FTH1 is involved in iron storage, while GPX4 plays a role in inhibiting lipid peroxidation. Reduced expression of both indicates increased cellular susceptibility to ferroptosis. However, intervention with CSP, SSa, and NGN restored their expression, further proving the efficacy of these drugs in preventing ferroptosis [58, 59].

Although the suppressive impacts of CSP, SSa, and NGN on autophagy and ferroptosis have been confirmed, the complex connection between autophagy and ferroptosis in this model is still unclear. To understand the complex connection between autophagy and ferroptosis in our NASH model, we utilized 3-MA, a widely recognized inhibitor of autophagy [60]. The findings were enlightening, highlighting the interconnectedness of these two procedures within the framework of NASH. TEM vividly captured the stark alterations in mitochondrial morphology in NASH-afflicted mice. The characteristic mitochondrial derangements, typified by a disrupted architecture and diminished mitochondrial cristae, were telltale signs of ongoing ferroptosis. Interestingly, 3-MA intervention significantly improved these mitochondrial abnormalities, indicating the protective effect of inhibiting autophagy against ferroptosis driven by mitochondria. In terms of biochemistry, 3-MA decreased the increased levels of ROS, Fe^2+^, and MDA observed in NASH while enhancing the reduced antioxidative indicators SOD and GSH-PX. At the molecular level, the increase in KEAP1 and the decreases in Nrf-2, FTH1, and GPX4 caused by NASH were reversed when 3-MA intervention was applied, emphasizing its protective function against ferroptosis. These findings support the hypothesis that strategies targeting autophagy could be harnessed as therapeutic modalities to counteract ferroptosis-driven liver injury in NASH.

**Figure 7:**
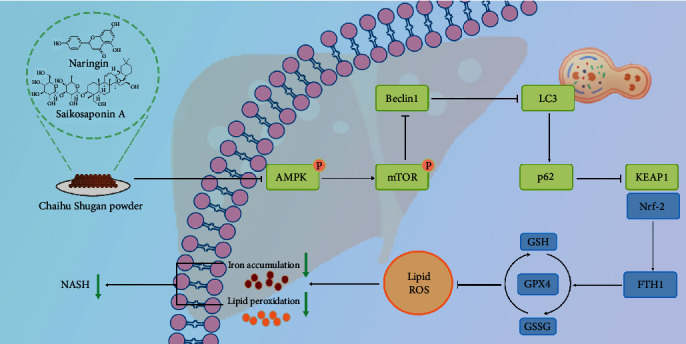
The AMPK–mTOR pathway is regulated by CSP, SSa, and NGN to attenuate hepatic steatosis, hepatocyte injury, and inflammation in NASH by controlling autophagy-induced ferroptosis. AMPK–mTOR, Adenosine monophosphate-activated protein kinase–mammalian target of rapamycin; CSP, Chaihu Shugan powder; NASH, nonalcoholic steatohepatitis; NGN, naringin; ROS, reactive oxygen species; SSa, Saikosaponin A.

Figure 7 illustrates how CSP, SSa, and NGN alleviate NASH by inhibiting autophagic ferroptosis through the AMPK–mTOR pathway.

## 5. Conclusions

Through the AMPK–mTOR pathway, this research has shown that CSP has the ability to alleviate NASH by suppressing autophagic ferroptosis. The ability of CSP to block AMPK and activate mTOR helps prevent excessive cell death in liver cells, which plays a crucial role in the progression of NASH. These findings highlight a promising therapeutic role for CSP in NASH, suggesting that modulating the autophagic ferroptosis pathway could be an effective treatment strategy.

## Data Availability

The original data used to support the findings of this study are available from the author upon request.

## Supporting Information

Supporting Information 1 The supporting information for this study include the HPLC chromatogram and “Author Checklist-E10.” Based on the HPLC chromatograms, SSa and NGN were detected in CSP. “Author Checklist-E10” adheres to the ARRIVE Essential 10 guidelines. These guidelines represent the foundational requirements for reporting animal research.

Supporting Information 2 HPLC chromatograms. (a and b) CSP contains both SSa and NGN.
