# Supplementary material for: The AMPK–mTOR Pathway Is Inhibited by Chaihu Shugan Powder, Which Relieves Nonalcoholic Steatohepatitis by Suppressing Autophagic Ferroptosis
Source: Mediators Inflamm. 2024 Oct 28;2024:4777789. doi: 10.1155/2024/4777789 (PMC11535263; doi:10.1155/2024/4777789)
Supplement: Supporting Information 2 — HPLC chromatograms. (a and b) CSP contains both SSa and NGN. [file 4777789.f2.pdf]

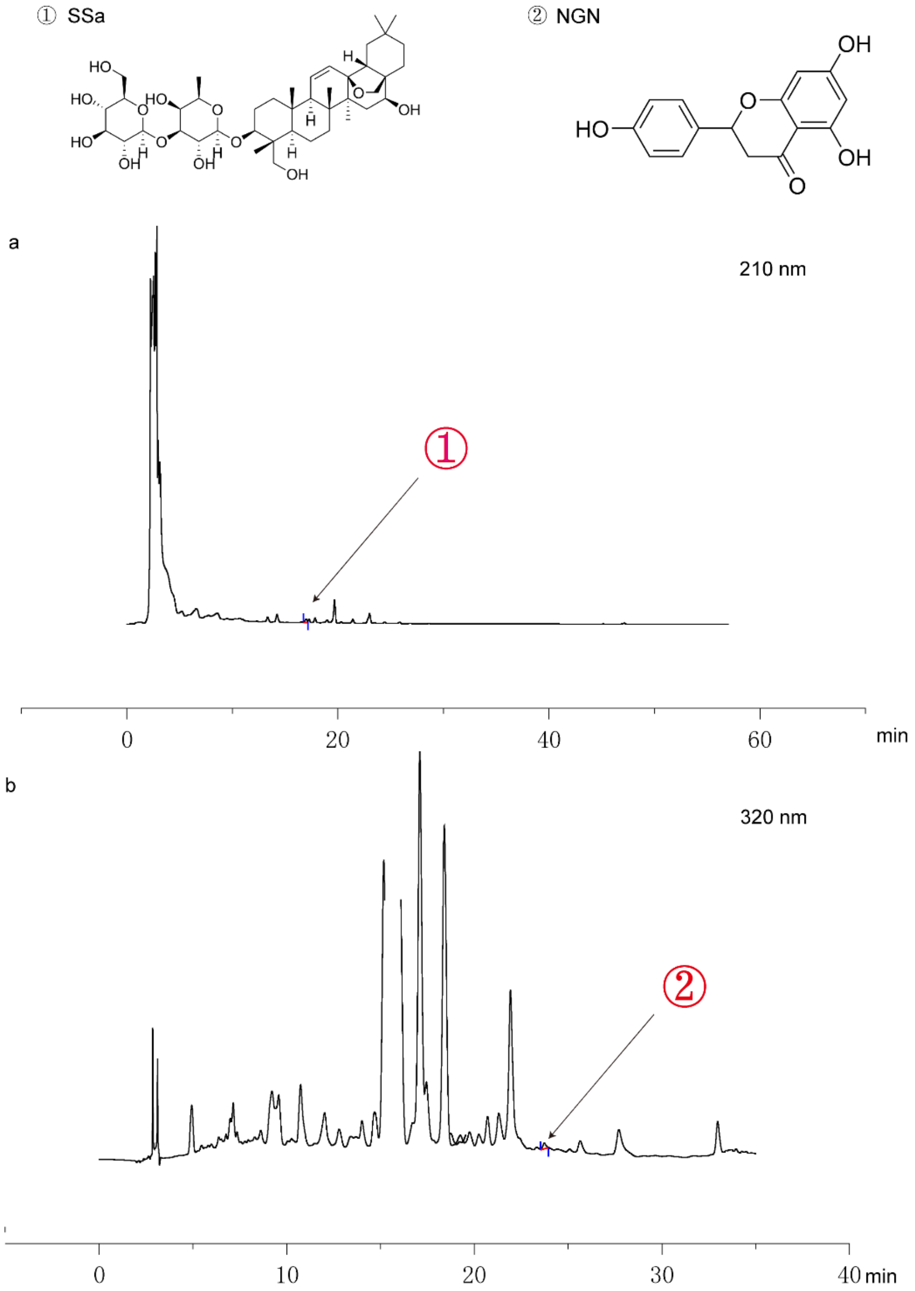

Figure S1: HPLC chromatograms. **(a–b)** CSP contains both SSa and NGN.

S1. SSa and NGN were detected in CSP

Based on the HPLC chromatograms, the peaks corresponding to SSa and NGN were identified, with retention times of 16.985 and 23.727 minutes, respectively (refer to Figure S1 a–b).
